# Cytotoxic Function and Cytokine Production of Natural Killer Cells and Natural Killer T-Like Cells in Systemic Lupus Erythematosis Regulation with Interleukin-15

**DOI:** 10.1155/2019/4236562

**Published:** 2019-03-31

**Authors:** Syh-Jae Lin, Ming-Ling Kuo, Hsiu-Shan Hsiao, Pei-Tzu Lee, Wen-I Lee, Ji-Yih Chen, Jing-Long Huang

**Affiliations:** ^1^Department of Pediatrics, Division of Asthma, Allergy, and Rheumatology, Chang Gung Memorial Hospital, College of Medicine, Chang Gung University, Taoyuan, Taiwan; ^2^Department of Microbiology and Immunology, Graduate Institute of Biomedical Sciences, College of Medicine, Chang Gung University, Tao-Yuan, Taiwan; ^3^Department of Medicine, Division of Allergy, Immunology and Rheumatology, Chang Gung Memorial Hospital, Chang Gung University College of Medicine, Tao-Yuan, Taiwan

## Abstract

Natural killer cells and NKT-like cells are the first line immune defense against tumor and virus infection. Deficient NK and NKT-like cell effector function may contribute to increased susceptibility to infection in SLE patients. We sought to examine the perforin and granzyme B expression, interferon-gamma (IFN-*γ*), and tumor-necrosis factor-alpha (TNF-*α*) production and CD107a degranulation of NK and NKT-like cells from SLE patients and their regulation by IL-15. We established that (1) perforin expression on SLE NK cells was decreased but unrelated to disease activity; (2) the MFI of granzyme B was increased in NK cells from SLE patients with active disease, associated with increased percentages of granzyme B^+^ CD56^bright^ NK cells; (3) NK cells from active SLE patients, both CD56^dim^ and CD56^bright^ NK subsets, produced higher IFN-*γ* compared to controls; (4) CD56^dim^, but not CD56^bright^ NK cells from active SLE patients, produced lower TNF-*α*, compared to inactive SLE patients and controls; (5) CD107a degranulation of SLE NK cells was comparable to controls; (6) IL-15 enhanced perforin/granzyme B expression, IFN-*γ*/TNF-*α* production, and CD107a degranulation of NK cells from SLE patients; and (7) similar observations were found for CD56^+^CD3^+^ NKT-like cells. Taken together, we demonstrated the differential expression of the heightened granzyme B and decreased TNF-*α* in NK and NKT-like cells in SLE patients. Higher granzyme B expression of NK and NKT-like cells in active SLE patients, further enhanced by circulating IL-15, may contribute to the maintenance of inflammation in SLE.

## 1. Introduction

Natural killer (NK) cells are a distinct lineage of CD3^−^, CD16^+^, and/or CD56^+^ lymphoid cells capable of killing tumor target without prior sensitization and produce various cytokines and chemokines which amplify an inflammatory response [1, 2]. The NK cells consist of two subsets: CD56^dim^ CD16^+^ NK subset which is more cytotoxic and CD56^bright^ CD16^−^ subset which produces abundant cytokines and plays an important immunoregulatory role [3, 4].

CD3^+^CD56^+^ NKT-like cells are a broad group of CD3^+^ T-cells coexpressing T-cell antigen receptor (TCR) and NK cell markers, thus possessing both innate and acquired immune functions [5, 6]. Like NK cells, NKT-like cells can secrete cytotoxic enzymes and cytokines to kill target cells in a non-MHC-restricted fashion. CD3^+^CD56^+^ NKT-like cells have been demonstrated to play an important role in antitumor and antivirus immune response [7, 8].

Systemic lupus erythematosus (SLE) is an autoimmune disease with multiorgan involvement caused by many immunologic abnormalities of the immune cells [9, 10]. The hallmarks of SLE are the production of various autoantibodies against self-antigens. Immune-complex deposition causes tissue damage and may lead to immune cell activation [11]. Previous works including ours have examined the various functional and numeric deficiencies of NK cells [4, 12, 13] and NKT-like cells [14, 15] from SLE patients.

In the present study, we investigated the cytotoxic function (perforin/granzyme B expression and CD107a degranulation) and cytokine production (IFN-*γ* and TNF-*α*) of NK (including NK^dim^ and NK^bright^ subsets) and NKT-like cells from SLE patients in various disease statuses. We also sought to determine whether IL-15, an NK-enhancing cytokine, would differentially affect their expression in SLE and healthy controls.

## 2. Methods

### 2.1. Study Subject

36 SLE patients were recruited from the Division of Asthma, Allergy, and Rheumatology, Department of Pediatrics, and the Division of Rheumatology and Immunology, Department of Internal Medicine, Chang Gung Memorial Hospital, between January 2014 and June 2016. 19 age-matched normal controls (male : female = 3 : 16) who did not have autoimmune disease, were free from active infection, and were also recruited for comparison. The diagnosis of SLE fulfills the 1997 American College of Rheumatology classification criteria [16].

The patients were evaluated using SLE disease activity index (SLEDAI) [17] and classified as having active disease if the score was greater than 6. Their medication history and laboratory findings were obtained from thorough chart reviews. All patients and controls were provided informed consent, and the study protocol was approved by the Ethics Committees of the hospital.

### 2.2. Cell Preparation

Peripheral blood was drawn from SLE patients and normal controls according to the guidelines of the Human Subjects Protection Committee of the hospital. The samples were collected in sterile tubes containing heparin and processed within 24 hours. Mononuclear cells (MNCs) were separated by Ficoll-Hypaque density gradient centrifugation and then incubated in RPMI-1640 containing 10% fetal calf serum. MNCs were stimulated with or without IL-15 (10 ng/mL, Peprotech, Rocky Hill, USA) for 18 hours.

### 2.3. Flow Cytometric Analysis of Perforin and Granzyme B Expression

MNCs were stained with anti-CD3 allophycocyanin (APC) and anti-CD56 fluorescein isothiocyanate- (FITC-) conjugated mouse anti-human monoclonal antibodies, from Becton-Dickinson (BD Pharmingen, San Diego, CA, USA) as previously described [4]. Intracellular perforin and granzyme B expressions in NK cells were investigated after permeabilization of the cell membrane with a Cytofix/Cytoperm kit (BD Pharmingen, San Diego, CA, USA) using PE or PerCP-conjugated anti-perforin or anti-granzyme B (BD Pharmingen, San Diego, CA, USA), according to the manufacturers' instructions.

**Figure 1:**
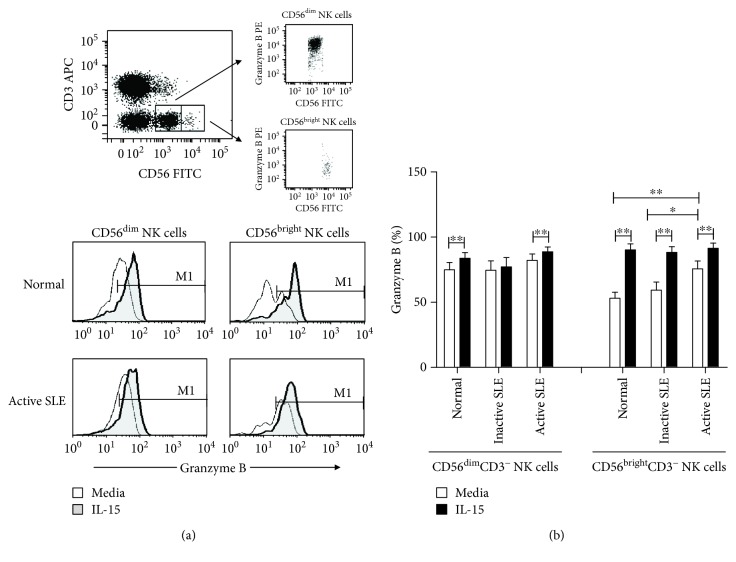
Granzyme B expression in CD56^dim^ and CD56^bright^ NK cells. (a) A representative dotplot showing the gating of CD56^dim^ and CD56^bright^ NK cells stained with granzyme (above) and a representative histogram showing granzyme B expression in normal controls (normal) and SLE patients with active disease (active SLE) under the influence of IL-15 (below). (b) Comparison of the percentages of granzyme B expressing CD56^dim^ and CD56^bright^ NK cells among normal controls (normal), SLE patients with inactive disease (inactive SLE), and SLE patients with active disease.

The fluorescent staining was analyzed on a FACSCalibur (BD Biosciences) flow cytometer. Appropriate fluorochrome-conjugated isotype-matched controls were used for background staining. Each analysis was performed using at least 10,000 cells that were gated in the viable lymphocyte population, as determined by forward scatter versus side scatter properties. The lymphocytes were gated to identify CD3^+^and CD3^−^ cells for further analysis of the expression levels of CD56. The NK cells were defined as CD3^−^ and CD56^+^. As shown in Figure 1(a), the two subpopulations of NK cells, CD56^dim^ and CD56^bright^ NK subsets, could be further defined according to the density of expression of CD56.

### 2.4. Measurement of IFN-*γ* and TNF-*α* Protein Expression

MNCs (10^6^ cells/well) were incubated in complete RPMI 1640 culture medium in the presence of 50 ng/mL phorbol myristate acetate (PMA; Sigma-Aldrich, St. Louis, MO, USA) and 1.0 *μ*g/mL ionomycin (Sigma-Aldrich, St. Louis, MO, USA) for 2 h at 37°C in 5% CO_2_. Thereafter, brefeldin A (GolgiPlug, BD Pharmingen, San Diego, CA, USA) was added to the cultures, which were incubated for 4 h. After incubation for 6 h, MNCs were then washed with 2 mL of cold PBS and resuspended in cytometry buffer. Surface staining with anti-CD56 and anti-CD3 mAbs was performed. For intracellular staining of IFN-*γ* and TNF-*α*, MNCs were fixed with 100 *μ*L of 4% paraformaldehyde (30 min at room temperature) and then permeabilized with 300 *μ*L of 0.5% saponin plus 10% fetal bovine serum in PBS (30 min at room temperature). After several washes, MNCs were stained with anti-IFN-*γ* PE (clone 4SB3, BD Pharmingen, San Diego, CA, USA) or anti-TNF-*α* PE (clone MAB11, BD Pharmingen, San Diego, CA, USA) (30 min at room temperature). Finally, MNCs were washed with cytometry buffer and stored at 4°C until analysis.

### 2.5. CD107a Degranulation Assay

The degranulation assay was performed as described elsewhere [18], with minor modifications. MNCs were stained with anti-CD107a PE and anti-CD3/CD56 allophycocyanin- (APC-) or fluorescein isothiocyanate- (FITC-) conjugated mouse anti-human monoclonal antibodies (APC/FITC) (BD Pharmingen, San Diego, CA, USA) and incubated for 25 minutes at 37°C in the dark. In some experiments, MNCs were cocultured for 4 hours with K562 cells at ratios of 12.5 : 1. CD107a expression on CD3^−^CD56^+^ NK cells and CD3^+^CD56^+^ NKT-like cells were then analyzed on a FACSCalibur flow cytometer, respectively.

### 2.6. Statistical Analysis

The data are presented as the means ± standard error of the mean. The nonparametric Wilcoxon signed rank test was applied for the analysis of the responses before and after IL-15 treatment. The Mann-Whitney *U* test was used to compare SLE and healthy donor responses. *p* values less than 0.05 were considered statistically significant. SPSS 9.0 software was used to perform the analysis.

## 3. Result

### 3.1. Demographic Characteristics of SLE Patients

The clinical characteristics of SLE patients are shown in Table 1. Patients were predominantly female (age between 12 and 69 years). Patients with active SLE disease had lower C3 and C4, higher level of anti-dsDNA, and higher percentages of nephritis and received higher doses of prednisolone.

**Figure 2:**
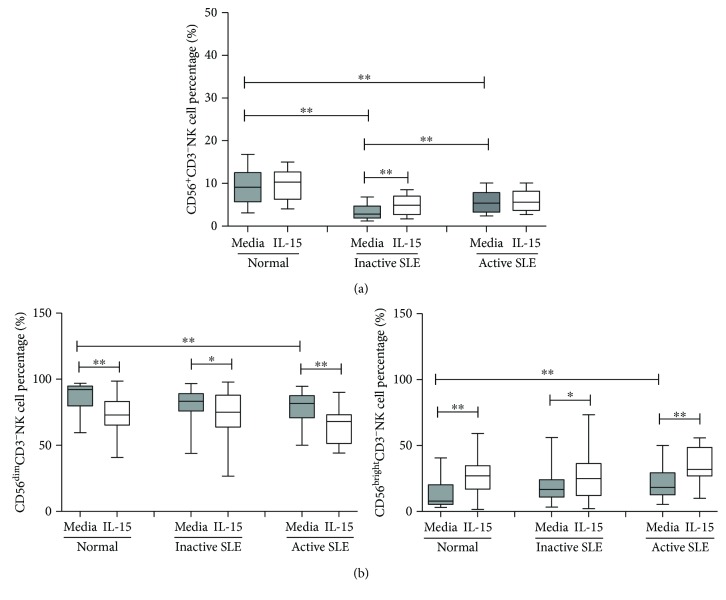
The percentage of NK cells from peripheral blood of SLE patients and healthy normal controls under the influence of IL-15. (a) Total NK cells. (b) CD56^dim^ NK and CD56^bright^ NK cells. MNCs were stimulated with or without IL-15 (10 ng/mL) for 18 hrs. Cells were stained by anti-CD3 and anti-CD56 antibodies and quantified by flow cytometry. Data was expressed as percent expression (%) ± SEM. ^∗^ *p* < 0.05 and ^∗∗^ *p* < 0.01 (normal control, *n* = 19; SLE with inactive disease, *n* = 15; SLE with active disease, *n* = 21).

The percentages of CD56^+^CD3^−^NK cells from overall SLE patients were lower than those from controls (4.7 ± 0.4% vs. 9.1 ± 1.0%, *p* < 0.001). As shown in Figure 2(a), the percentages of NK cells were higher in SLE patients with active disease than those with inactive SLE disease (5.6 ± 0.6% vs. 3.5 ± 0.5%, *p* = 0.009). IL-15 increased the percentages of NK cells in SLE patients with inactive disease (4.9 ± 0.6% vs. 3.5 ± 0.5%, *p* = 0.001), but not SLE patients with active disease (5.9 ± 0.5% vs. 5.6 ± 0.6, *p* = 0.104). When further subdivided into CD56^bright^ NK and CD56^dim^ NK cells, SLE NK cells contained more CD56^bright^ NK cells (19.7 ± 2.1% vs. 12.3 ± 2.5%, *p* = 0.010) and fewer CD56^dim^ NK cells compared to controls (80.3 ± 2.1% vs. 87.7 ± 2.5%, *p* = 0.010). IL-15 decreased the percentages of CD56^dim^ NK cells while increasing the CD56^bright^ NK cells (Figure 2(b)).

### 3.2. Perforin and Granzyme B Expression on NK Cells

**Figure 3:**
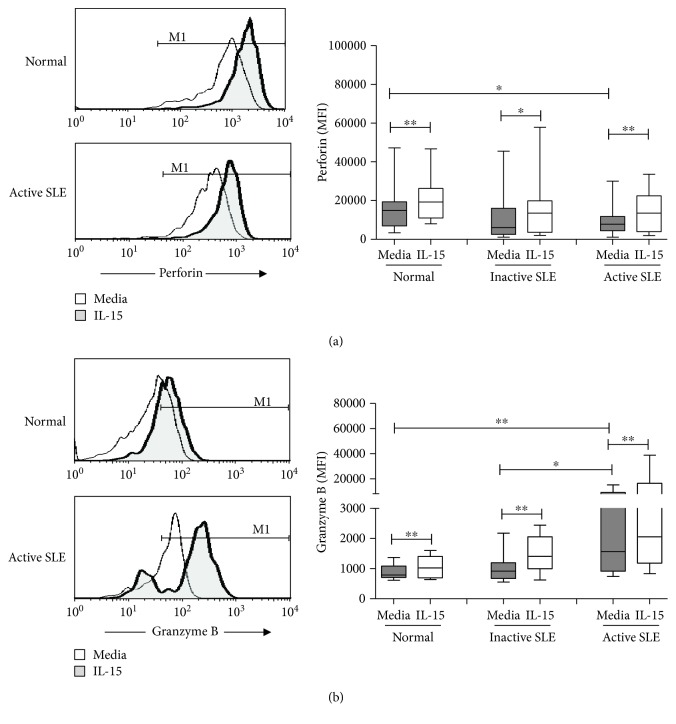
Perforin (a) and granzyme B (b) expression of NK cells from SLE and healthy controls under the influence of IL-15. A representative histogram comparing normal controls (normal) and SLE patients with active disease (active SLE) under the influence of IL-15 is shown (left). Comparison was also made among normal controls (normal), SLE patients with inactive disease (inactive SLE), and SLE patients with active disease (active SLE). PBMC were stimulated with or without IL-15 (10 ng/mL) for 18 hrs. Data was expressed as mean fluorescence intensity (MFI) ± SEM of positive cells. ^∗^ *p* < 0.05 and ^∗∗^ *p* < 0.01 (normal control, *n* = 17; SLE with inactive disease, *n* = 14; SLE with active disease, *n* = 17).

Figure 3 shows the perforin and granzyme B expression on NK cells from controls, inactive SLE patients, and active SLE patients. The mean fluorescence intensity (MFI) was used to quantify NK cells stained positive for perforin or granzyme B. The MFI of perforin was lower in NK cells from active SLE NK cells compared to that in controls (9052 ± 1696 vs. 15640 ± 2645, *p* = 0.031). No difference of perforin MFI was found between patients with active and inactive disease (9052 ± 1696 vs. 12208 ± 3909, *p* = 0.662). Similar to controls, IL-15 enhanced the MFI of perforin in NK cells from SLE patients irrespective of disease status.

As shown in Figure 3(b), the MFI of granzyme B was higher in active SLE NK cells compared to that in controls (5274 ± 1453 vs. 890 ± 64, *p* = 0.003). SLE patients with active disease also have higher granzyme B expression compared to those with inactive disease (5274 ± 1453 vs. 1024 ± 138, *p* = 0.031). No difference could be found between controls and inactive SLE patients (890 ± 64 vs. 1024 ± 138, *p* = 0.511). IL-15 enhanced the granzyme B expression of NK cells from controls, inactive SLE patients, and active SLE patients, respectively.

NK cells were further divided into CD56^dim^ and CD56^bright^ subsets (Figure 1). Greater than 95% of NK cells expressed perforin (data not shown). The percentages of granzyme B^+^ NK cells were higher in CD56^dim^ subsets than in CD56^bright^ subsets. The percentages of granzyme B^+^ CD56^bright^ NK cells were higher in SLE patients with active disease compared to those in controls (75.7 ± 5.9% vs. 53.1 ± 4.7%, *p* = 0.002) and to SLE patients with inactive disease (75.7 ± 5.9% vs. 59.3 ± 6.2%, *p* = 0.024), respectively. IL-15 increased the percentages of granzyme B^+^ NK cells from both CD56^dim^ and CD56^bright^ NK subsets, with CD56^bright^ NK subsets responding better to IL-15 stimulation than CD56^dim^ subsets.

### 3.3. IFN-*γ* and TNF-*α* Production of NK Cells

**Figure 4:**
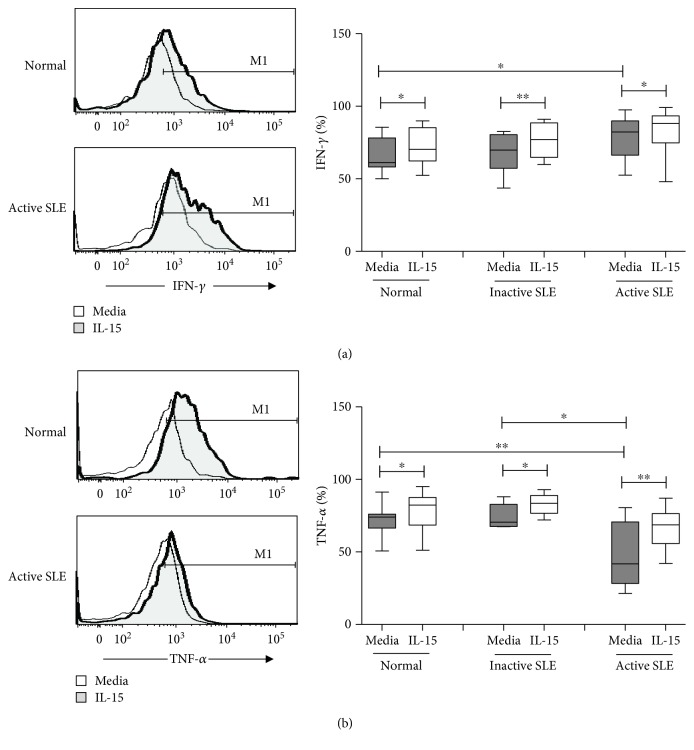
IFN-*γ* (a) and TNF-*α* (b) expression of NK cells from SLE and healthy controls under the influence of IL-15. A representative histogram comparing normal controls (normal) and SLE patients with active disease (active SLE) under the influence of IL-15 is shown (left). Comparison was also made among normal controls (normal), SLE patients with inactive disease (inactive SLE), and SLE patients with active disease (active SLE). PBMC were stimulated with or without IL-15 (10 ng/mL) for 18 hrs. Data was expressed as percent expression (%) ± SEM. ^∗^ *p* < 0.05 and ^∗∗^ *p* < 0.01 (Normal control, *n* = 17; SLE with inactive disease, *n* = 10; SLE with active disease, *n* = 11).

Figure 4 shows IFN-*γ* and TNF-*α* production of NK cells from SLE and controls. NK cells from active SLE patients produced higher IFN-*γ* compared to those from controls (79.5 ± 4.3% vs. 67.2 ± 2.8%, *p* = 0.017). NK cells from inactive SLE patients produced comparable IFN-*γ* compared to those from controls (68.3 ± 4.2% vs. 67.2 ± 2.8%, *p* = 0.651). IL-15 enhanced IFN-*γ* production of SLE NK cells and controls alike (Figure 4(a)).

As shown in Figure 4(b), the production of TNF-*α* of NK cells from active SLE patients was lower compared to that from controls (48.0 ± 6.4% vs. 73.1 ± 2.4%, *p* = 0.003) and SLE patients with inactive disease (48.0 ± 6.4% vs. 74.2 ± 3.5%, *p* = 0.021), respectively. The production of TNF-*α* of NK cells from inactive SLE patients was comparable to that from controls (74.2 ± 3.5% vs. 73.1 ± 2.4%, *p* = 0.883). IL-15 enhanced TNF-*α* production of SLE NK cells to a similar degree observed with controls.

IFN-*γ* and TNF-*α* production on CD56^dim^ NK cells and CD56^bright^ NK cells under the influence of IL-15 is shown in Table 2. Both CD56^dim^ and CD56^bright^ NK cells produced IFN-*γ*. The level of IFN-*γ* production did not differ between CD56^dim^ and CD56^bright^ subsets in SLE patients and was unrelated to disease activity.

CD56^dim^ NK cells from active SLE patients expressed lower percentages of TNF-*α* compared to those from controls (46.9 ± 6.6% vs. 73.7 ± 2.5%, *p* = 0.001), while the percentages of TNF-*α* expressing CD56^bright^ NK cells did not differ (73.7 ± 7.6% vs. 73.0 ± 2.7%, *p* = 0.812). Patients with active SLE disease also had lower percentages of CD56^dim^ NK cells expressing TNF-*α* than those with inactive disease (46.9 ± 6.6% vs. 75 ± 3.5%, *p* = 0.009). IL-15 enhanced the TNF-*α* expression of CD56^dim^ NK cells and CD56^bright^ NK cells from SLE patients and controls alike.

### 3.4. Perforin and Granzyme B Expression on NKT-Like Cells

**Figure 5:**
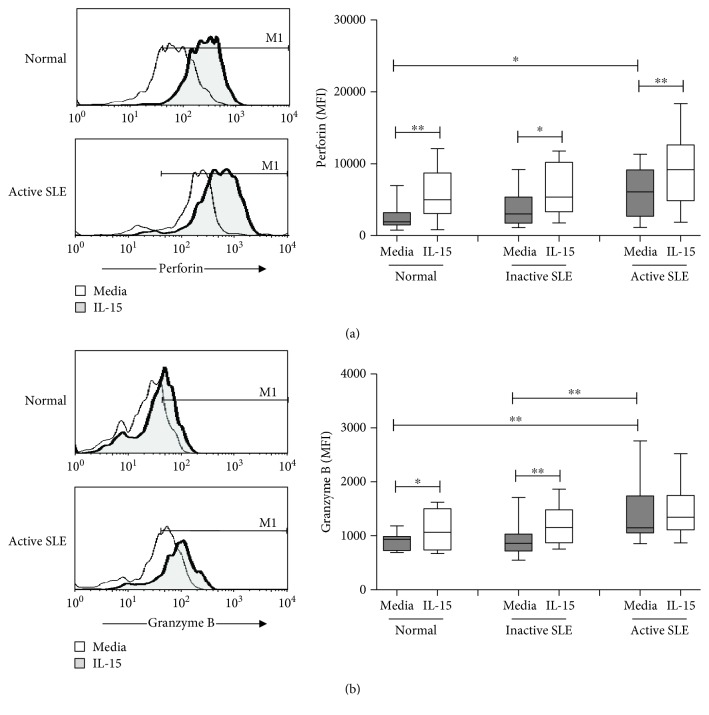
Perforin (a) and granzyme B (b) expression of NKT-like cells from SLE and healthy controls under the influence of IL-15. A representative histogram comparing normal controls (normal) and SLE patients with active disease (active SLE) under the influence of IL-15 is shown (left). Comparison was also made among normal controls (normal), SLE patients with inactive disease (inactive SLE), and SLE patients with active disease (active SLE). PBMC were stimulated with or without IL-15 (10 ng/mL) for 18 hrs. Data was expressed as mean fluorescence intensity (MFI) ± SEM of positive cells. ^∗^ *p* < 0.05 and ^∗∗^ *p* < 0.01 (normal control, *n* = 12; SLE with inactive disease, *n* = 13; SLE with active disease, *n* = 14).

Figure 5 shows the perforin and granzyme B expression on CD3^+^CD56^+^ NKT-like cells from SLE and controls. NKT-like cells from active SLE expressed higher perforin levels compared to those from controls (6192 ± 893 vs. 2735 ± 591, *p* = 0.01). There was no significant difference in perforin MFI of NKT-like cells between SLE patients with active and inactive diseases (6192 ± 893 vs. 3743 ± 679, *p* = 0.052). IL-15 enhanced perforin MFI of SLE NK cells to a similar degree observed with controls.

The expression of granzyme B on NKT-like cells from active SLE patients was higher than that from controls (1395 ± 124 vs. 889 ± 47, *p* = 0.001). The MFI of granzyme B of NKT-like cells was also higher in active SLE disease than in inactive disease (1395 ± 124 vs. 913 ± 72, *p* = 0.001). Similar to NK cells, IL-15 enhanced both perforin and granzyme B expressions of NKT-like cells from controls and inactive SLE patients, respectively. IL-15 enhanced perforin, but not granzyme B expression, of NKT-like cells from SLE with active disease.

### 3.5. IFN-*γ* and TNF-*α* Production of NKT-Like Cells

**Figure 6:**
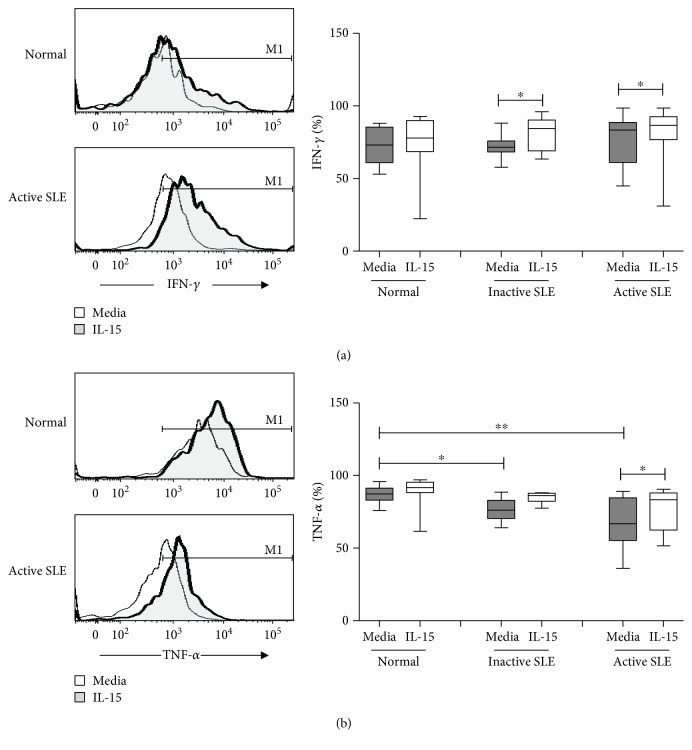
IFN-*γ* (a) and TNF-*α* (b) expression of NKT-like cells from SLE and healthy controls under the influence of IL-15. A representative histogram comparing normal controls (normal) and SLE patients with active disease (active SLE) under the influence of IL-15 is shown (left). Comparison was also made among normal controls (normal), SLE patients with inactive disease (inactive SLE), and SLE patients with active disease (active SLE). PBMC were stimulated with or without IL-15 (10 ng/mL) for 18 hrs. Data was expressed as percent expression (%) ± SEM. ^∗^ *p* < 0.05 and ^∗∗^ *p* < 0.01 (normal control, *n* = 14; SLE with inactive disease, *n* = 9; SLE with active disease, *n* = 13).

Figure 6 shows the IFN-*γ* and TNF-*α* production of NKT-like cells from SLE and controls. Similar to that observed with NK cells, NKT-like cells from active SLE produced comparable IFN-*γ* compared to those from controls (77.0 ± 5.0% vs. 72.2 ± 3.1%, *p* = 0.244). There was no significant difference in IFN-*γ* expression of NKT-like cells from SLE patients with active and inactive disease (77.0 ± 5.0% vs. 72.1 ± 2.7%, *p* = 0.193). Compared to controls, TNF-*α* expression of NKT-like cells was lower in both inactive (76.4 ± 3.4% vs. 86.8 ± 1.7%, *p* = 0.017) and active (65.7 ± 5.4% vs. 86.8 ± 1.7%, *p* = 0.002) SLE patients. TNF-*α* expression of NKT-like cells did not differ between active SLE disease and inactive disease (65.7 ± 5.4% vs. 76.4 ± 3.4%, *p* = 0.191).

### 3.6. CD107a Degranulation of NK Cells and NKT-Like Cells

**Figure 7:**
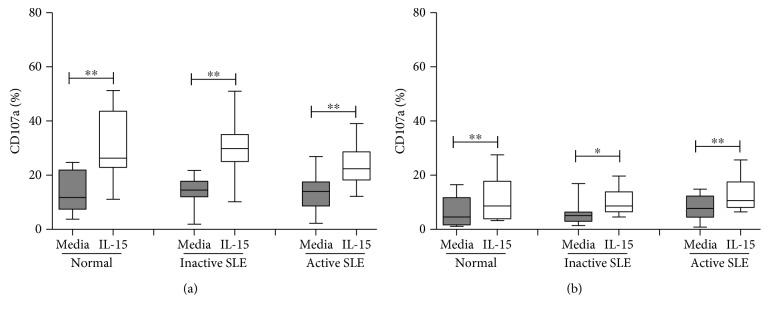
CD107a expression of (a). CD56^+^CD3^−^ NK cells and (b) CD56^+^CD3^+^ NKT-like cells from SLE and healthy controls under the influence of IL-15. Comparison was also made among normal controls (normal), SLE patients with inactive disease (inactive SLE), and SLE patients with active disease (active SLE). PBMC were stimulated with or without IL-15 (10 ng/mL) for 18 hrs. Data was expressed as percent expression (%) ± SEM. ^∗^ *p* < 0.05 and ^∗∗^ *p* < 0.01 (normal control, *n* = 12; SLE with inactive disease, *n* = 14; SLE with active disease, *n* = 18).

CD107a expression of NK and NKT-like cells following contact with K562 cells is shown in Figure 7. The level of CD107a expression on NK cells following contact of K562 cells was comparable to controls in both inactive (14.1 ± 1.3% vs. 13.4 ± 2.2%, *p* = 0.797) and active (13.1 ± 1.6% vs. 13.4 ± 2.2%, *p* = 0.982) SLE patients (Figure 7(a)). No difference of CD107a expression on NK cells was found between SLE patients with active disease and inactive disease. IL-15 enhanced CD107a expression of NK cells from SLE patients following contact with K562 cells, similar to that observed in controls.

Similar to NK cells, SLE NKT-like cells have comparable CD107a expression compared to controls irrespective of disease status. CD107a expression on NKT-like cells was not significantly different between SLE patients with active disease and inactive diseases (7.8 ± 1.0% vs. 5.8 ± 1.2%, *p* = 0.180).

## 4. Discussion

We have previously demonstrated the dysfunctional NK and NKT-like cells in SLE patients with regard to CD11b and CD62L expression and their response to IL-15 [14]. Our recent study has also characterized the expression of NCR and iNKR on NK cells in SLE patients in relation to their disease activity [4]. In the present study, we sought to examine the cytotoxic machinery (perforin/granzyme B expression and CD107a degranulation), as well as cytokine production (IFN-*γ* and TNF-*α* production) in NK and NKT-like cells from SLE patients and their regulation with IL-15.

Perforin is a 70 kDa glycoprotein responsible for pore formation in the cell membrane of target cells induced by NK cells [19]. Granzyme B is a serine protease found in the cytoplasmic granules of NK cells that triggers apoptotic changes of target cells [20]. Both are important mediators responsible for NK cytotoxicity. Previous studies regarding perforin and granzyme B expression of NK cell in SLE patients yielded contradictory results. Park et al. showed that the NK cytotoxicity is decreased due to the deficient granzyme B and perforin expression [21]. However, Henriques et al. revealed that the percentage of NK cells expressing granzyme B and perforin was higher, particularly in SLE with active disease [22]. We found decreased perforin yet increased granzyme B expression in SLE patients with active disease. The percentages of CD56^bright^ NK cells expressing granzyme B were higher in SLE patients with active disease. Our finding is in agreement with Shah et al.'s showing that soluble granzyme B correlated with SLE disease activity [23]. Kok et al. showed that granzyme B may contribute to the pathogenesis of lupus nephritis [24].

We observed an increase of the percentages of IFN-*γ* ^+^ NK cells in SLE with active disease, which could be observed in both CD56^dim^ and CD56^bright^ NK subsets. Our finding was consistent with Hervier et al.'s who reported the enhanced frequency of IFN-*γ* ^+^ NK cells in patients with active SLE [25] and suggests that Th1 cytokines like IFN-*γ* may play a pathogenic role in SLE.

TNF-*α* is a potent inflammatory mediator and apoptosis inducer. Previous studies have shown that TNF-*α* gene polymorphism was involved in the susceptibility of SLE [26] and increased serum levels of TNF-*α* are observed in SLE patients [27, 28]. However, we found that NK cells from SLE patients produce lower TNF-*α*, especially in patients with active disease. We found decreased percentages of TNF-*α*+NK cells in CD56^dim^, but not CD56^bright^, subsets from active SLE subjects, in contrast to Henriques et al.'s who showed both CD56^dim^ and CD56^bright^ NK cells were deficient [22]. This may be due to an intrinsic defect of CD56^dim^ NK cells in active SLE patients, or CD56^dim^ NK cells expressing TNF-*α* might have migrated into target organs [12]. Gómez et al. showed that the TNF-*α* levels were higher in SLE patients with inactive disease compared with patients with active disease, suggesting that TNF-*α* may be protective in SLE [29]. Anti-TNF-*α* therapy is still controversial in SLE as it may induce antinuclear antibodies and anti-ds-DNA [30].

Numerous studies including ours have confirmed reduced NK cytotoxicity in SLE patients [4, 21, 25]. The ability of NK cells to degranulate appears to be not correlated with their cytotoxicity. Hervier et al. show only slightly lower percentages of CD107a^+^ NK cells in active SLE disease while their cytotoxicity is severely impaired [25]. We found that the degranulatory ability of SLE NK cells after contract with K562 cells is intact, in agreement with Ye et al.'s [15].

The role of NKT-like (CD3^+^CD56^+^) cells in the pathogenesis of SLE is unclear. We have reported decreased numbers of circulating NKT-like cells in SLE patients [14]. In the present study, we found that NKT-like cells express lower levels of perforin and granzyme B per cell than corresponding NK cells, while their IFN-*γ* and TNF-*α* production is comparable. NKT-like cells behave similarly to NK cells with regard to perforin/granzyme B expression, cytokine production, and response to IL-15. We found that the CD107a expression of NKT-like cells in SLE was comparable to that in controls, in discrepancy with Ye et al.'s who showed that the percentages of stimulated CD107a^+^ NKT cells in SLE patients were significantly lower than those in the controls [15].

Our previous study has shown that SLE patients with active disease show higher IL-15 serum levels compared to inactive patients [4]. Aringer et al. showed that both lymphocyte CD25 and Bcl-2 expressions significantly correlated with serum IL-15 and were increased by IL-15 [31]. However, Baranda et al. showed that mononuclear cells from SLE patients responded poorly to IL-15 simulation [32]. We showed that IL-15 is able to enhance perforin/granzyme B and cytokine production of NK cells from SLE patients, similar to that observed in control NK cells. NKT-like cells from SLE patients also responded readily to IL-15. Therefore, overexpression of IL-15 in SLE patient did not render their NK or NKT-like cells refractory to subsequent IL-15 stimulation.

Bo et al. reported the association of high transmembrane IL-15 with murine lupus development [33]. Spada et al. demonstrated elevated IL-15/IL-15R complex levels in the kidneys of diseased MRL/MpJ^lpr^ mice [34]. We found that IL-15 further enhanced granzyme B expression in active SLE NK cells, suggesting that IL-15 may play a pathogenic role in patients with of SLE.

Taken together, we demonstrated the differential expression of heightened granzyme B and decreased TNF-*α* in NK and NKT-like cells in SLE patients. IL-15 may contribute to the maintenance of inflammation in active SLE by upregulating granzyme B and TNF-*α*.

## Figures and Tables

**Table 1:** Clinical characteristics of patients with systemic lupus erythematosis.

	Inactive SLE (*n* = 15)	Active SLE (*n* = 21)
Age (mean, range)	40.4 ± 1.7 (28-69)	33.8 ± 1.7 (12-68)
Disease duration (years)	6.8 ± 0.7	5.4 ± 0.6
SLEDAI (range)	0-6	7-25
C3 (mean ± SEM, mg/dL)	97.0 ± 9.3	65.3 ± 4.5
C4 (mean ± SEM, mg/dL)	23.3 ± 3.0	11.2 ± 1.7
Anti-ds DNA (mean ± SEM, mg/dL)	170.7 ± 34.1	370.0 ± 39.3
Malar rash (%)	29.2%	31.8%
Nephritis (%)	32.9%	51.6%
Arthritis (%)	71.8%	33.9%
Prednisolone (mg/day)	7.5 ± 1.1	12.5 ± 1.7
Azathioprine (%)	13.3%	30.0%
Mycophenolate mofetil (%)	5.0%	3.2%

**Table 2:** The percentages of IFN-*γ* and TNF-*α* expressing CD56^dim^ and CD56^bright^ NK cells in healthy controls (normal) and SLE patients with active and inactive disease.

			IFN-*γ* (%)	TNF-*α* (%)
CD56^dim^ NK cells	Normal	Media	67.7 ± 2.9	73.7 ± 2.5
		IL-15	73.6±2.9^∗∗^	78.5 ± 3.0^∗^
	Inactive SLE	Media	69.2 ± 3.8	75.0 ± 3.5
		IL-15	77.1±3.8^∗∗^	83.2 ± 3.0^∗^
	Active SLE	Media	78.5 ± 4.5^✝^	46.9 ± 6.6^✝✝,##^
		IL-15	84.4±3.9^∗∗^^,✝^	67.7±4.1^∗∗^^,✝,#^
CD56^bright^ NK cells	Normal	Media	70.3 ± 4.1	73.0 ± 2.7
		IL-15	80.1±4.0^∗∗^	84.1±3.7^∗∗^
	Inactive SLE	Media	69.4 ± 6.6	67.0 ± 7.0
		IL-15	72.2 ± 8.6	84.0 ± 2.2^∗^
	Active SLE	Media	78.0 ± 8.8	73.7 ± 7.6
		IL-15	89.0 ± 5.2^✝,#^	81.5 ± 8.1

^∗^ *p* < 0.05 and ^∗∗^ *p* < 0.01 compared to media; ^✝^ *p* < 0.05 and ^✝✝^ *p* < 0.01 compared to normal; ^#^ *p* < 0.05 and ^##^ *p* < 0.01 compared to inactive SLE.

## Data Availability

The data used to support the finding of this study are included within the supplementary information files.

## Supplementary Materials

Supplementary 1 Figure 1(b): comparison of the percentages of granzyme B expressing CD56^dim^ and CD56^bright^ NK cells among normal controls (normal), SLE patients with inactive disease (inactive SLE), and SLE patients with active disease (active SLE) in the presence and absence of IL-15.

Supplementary 2 Figure 2(a): comparison of the percentage of NK cells from peripheral blood of SLE patients (active and inactive) and healthy controls (normal) in the presence and absence of IL-15.

Supplementary 3 Figure 2(b): comparison of the percentage of CD56^dim^ and CD56^bright^ NK cells from peripheral blood of SLE patients (active and inactive) and healthy controls (normal) in the presence and absence of IL-15.

Supplementary 4 Figure 3(a): comparison of the MFI of perforin of NK cells from peripheral blood of SLE patients (active and inactive) and healthy controls (normal) in the presence and absence of IL-15.

Supplementary 5 Figure 3(b): comparison of the MFI of granzyme B of NK cells from peripheral blood of SLE patients (active and inactive) and healthy controls (normal) in the presence and absence of IL-15.

Supplementary 6 Figure 4(a): comparison of the percentages of IFN-*γ* expressing NK cells among normal controls (normal), SLE patients with inactive disease (inactive SLE), and SLE patients with active disease (active SLE) in the presence and absence of IL-15.

Supplementary 7 Figure 4(b): comparison of the percentages of TNF-*α* expressing NK cells among normal controls (normal), SLE patients with inactive disease (inactive SLE), and SLE patients with active disease (active SLE) in the presence and absence of IL-15.

Supplementary 8 Figure 5(a): comparison of the MFI of perforin of NKT-like cells from peripheral blood of SLE patients (active and inactive) and healthy controls (normal) in the presence and absence of IL-15.

Supplementary 9 Figure 5(b): comparison of the MFI of granzyme B of NKT-like cells from peripheral blood of SLE patients (active and inactive) and healthy controls (normal) in the presence and absence of IL-15.

Supplementary 10 Figure 6(a): comparison of the percentages of IFN-*γ* expressing NKT-like cells among normal controls (normal), SLE patients with inactive disease (inactive SLE), and SLE patients with active disease (active SLE) in the presence and absence of IL-15.

Supplementary 11 Figure 6(b): comparison of the percentages of TNF-*α* expressing NKT-like cells among normal controls (normal), SLE patients with inactive disease (inactive SLE), and SLE patients with active disease (active SLE) in the presence and absence of IL-15.

Supplementary 12 Figure 7(a): comparison of the percentages of CD107a expressing NK cells following contact with K562 cells among normal controls (normal), SLE patients with inactive disease (inactive SLE), and SLE patients with active disease (active SLE) in the presence and absence of IL-15.

Supplementary 13 Figure 7(b): comparison of the percentages of CD107a expressing NKT-like cells following contact with K562 cells among normal controls (normal), SLE patients with inactive disease (inactive SLE), and SLE patients with active disease (active SLE) in the presence and absence of IL-15.

Supplementary 14 Table 2: comparison of the percentages of IFN-*γ* and TNF-*α* expressing CD56^dim^ and CD56^bright^ NK cells in healthy controls (normal) and SLE patients with active and inactive disease in the presence and absence of IL-15.
