# Supplementary material for: Cytotoxic Function and Cytokine Production of Natural Killer Cells and Natural Killer T-Like Cells in Systemic Lupus Erythematosis Regulation with Interleukin-15
Source: Mediators Inflamm. 2019 Mar 31;2019:4236562. doi: 10.1155/2019/4236562 (PMC6462338; doi:10.1155/2019/4236562)
Supplement: Supplementary 1 — Figure 1(b): comparison of the percentages of granzyme B expressing CD56dim and CD56bright NK cells among normal controls (normal), SLE patients with inactive disease (inactive SLE), and SLE patients with active disease (active SLE) in the presence and absence of IL-15. [file 4236562.f1.pdf]

Figure 1(b)

Granzyme

CD56dim

Normal			Inactive SLE			Active SLE	
Media	IL-15		Media	IL-15		Media	IL-15
85	91.7		64.8	90.2		34.1	51.3
71.3	97.4		15.2	16.1		35	62.9
100	93.3		24.1	38.6		41.9	57.7
23	40.9		74.1	95.1		84	95.1
77.8	90.5		92.9	98.9		60.6	86.4
38.1	69.7		97.8	69.8		83.1	92.1
61.7	81.30		88.7	90.2		93.5	97.3
66.2	75.1		39.4	95.2		96.8	95.7
53.2	78.1		73.1	67.5		89.6	
61.3	63.5		65.6	38.7		80.8	72.6
64.0	50.9		95.5	60.9		87.4	97.9
82.5	95.2		97.8	98.7		91.4	99.1
98.8	99.7		98.9	99.6		98.3	99.9
98.7	99.9		96.4	99.6		97.7	99.2
97.2	98.8		93.6	99.2		98.8	99.9
97.5	98.3					97.8	99.7
97.7	99.8					76.5	81.5
						99.5	100
						99.7	100
						97	98.3

CD56bright							
Normal			Inactive SLE			Active SLE	
Media	IL-15		Media	IL-15		Media	IL-15
13.6	78.9		56.4	98.4		13.9	25
34.5	98.9		3.8	79.9		32.5	77.8
50.9	100		23.5	76.7		32.7	78.2
	23.1		58.3	88.2		76.6	96.5
31	94.4		64.3	100		34.8	95.8
27	75.9		83.2	91.5		81.6	94.5
39.8	92		47.8	97.3		76.6	97.8
64.1	95.5		32.5	93.5		90.3	90.6
54	96		61.7	50		61.1	
70.8	96.7		54.2	50		71.8	84.8
57.6	84.8		76.8	100		81.9	100
69.7	98.9		85.4	99.7		94.9	100
69.6	99.9		80.6	99.5		95	100
74.1	100		78.6	100		98.2	100
57.1	99.5		82.5	99.4		96.9	100
62	99.7					95.9	100
73.1	99.6					86.4	96.4
						100	100
						96.9	100
						96.1	100
