# Supplementary material for: Cytotoxic Function and Cytokine Production of Natural Killer Cells and Natural Killer T-Like Cells in Systemic Lupus Erythematosis Regulation with Interleukin-15
Source: Mediators Inflamm. 2019 Mar 31;2019:4236562. doi: 10.1155/2019/4236562 (PMC6462338; doi:10.1155/2019/4236562)
Supplement: Supplementary 2 — Figure 2(a): comparison of the percentage of NK cells from peripheral blood of SLE patients (active and inactive) and healthy controls (normal) in the presence and absence of IL-15. [file 4236562.f2.pdf]

Figure 2(a)

CD56+CD3- NK cell (%)

Normal			Inactive SLE			Active SLE	
Media	IL-15		Media	IL-15		Media	IL-15
9.1	11.6		3.2	5.7		5.4	4.9
10.4	10.7		1.2	2.3		3.2	3.8
3.1	5		5.6	7.1		7.5	8.3
6.5	13		2.3	4.6		4.6	5.6
7.4	6.3		1.9	2.6		8.2	9.1
7.5	6.9		2.7	2.7		3.9	5.1
11	12.7		2.8	3.0		9.1	10.1
16.8	13.1		1.5	1.7		2.4	3.2
15.8	10.3		4.4	5.1		2.4	2.7
4.1	4		6.8	8.5		7.6	8.1
3.1	4.3		6.4	8.3		2.4	2.8
4.5	4.4		2.4	3.9		7	6.5
13.2	14.9		1.7	7.0		2.9	3.6
5.7	6.6		4.3	5.5		4.7	4.5
7.8	8.5		4.7	4.9		9.6	8.4
9.2	10.4					3.3	3.4
11.3	9.5					10.1	8.7
12.5	10.3					3.4	4.3
14	15					8.1	7
						5.6	6.9
						6.2	6.7
