# Supplementary material for: Cytotoxic Function and Cytokine Production of Natural Killer Cells and Natural Killer T-Like Cells in Systemic Lupus Erythematosis Regulation with Interleukin-15
Source: Mediators Inflamm. 2019 Mar 31;2019:4236562. doi: 10.1155/2019/4236562 (PMC6462338; doi:10.1155/2019/4236562)
Supplement: Supplementary 3 — Figure 2(b): comparison of the percentage of CD56dim and CD56bright NK cells from peripheral blood of SLE patients (active and inactive) and healthy controls (normal) in the presence and absence of IL-15. [file 4236562.f3.pdf]

Figure 2(b)

CD56dim NK cell (%)

Normal			Inactive SLE			Active SLE	
Media	IL-15		Media	IL-15		Media	IL-15
96.7	89.7		75.9	69.4		94.7	80.7
72.6	75.0		82.4	40.1		70.8	44.2
94.2	92.5		80.6	68.9		84.2	50.6
96.9	98.5		88.1	90.3		81.7	54.7
75.7	66.7		82.1	65.9		78.4	51.4
88.0	53.6		86.0	79.3		72.1	73.1
76.2	70.9		68.8	26.7		92.9	84.0
94.6	76.4		75.9	57.4		68.2	79.8
59.5	49.6		96.7	87.0		50.0	66.7
81.0	40.8		92.1	97.8		67.8	67.0
93.3	88.6		84.3	83.1		69.9	46.8
95.5	67.1		87.2	87.2		81.8	72.0
91.2	66.7		94.7	92.3		87.5	90.1
93.6	69.4		43.9	70.9		76.9	71.4
92.4	79.8					91.2	72.3
89.4	61.1					89.3	68.5
92.0	76.7					87.5	65.6
96.4	81.3					80.4	50.7
						85.7	68.1

CD56bright NK cell (%)

Normal			Inactive SLE			Active SLE	
Media	IL-15		Media	IL-15		Media	IL-15
3.3	10.3		24.1	30.6		5.3	19.3
27.4	25.0		17.7	59.9		29.3	55.8
5.8	7.5		19.4	31.1		15.9	49.5
3.1	1.5		11.9	9.7		18.3	45.3
24.3	33.3		18.0	34.2		21.6	48.6
12.0	46.4		14.0	20.7		27.9	26.9
23.8	29.1		31.2	73.3		7.1	16.0
5.5	23.6		24.1	42.6		31.9	20.2
40.5	50.4		3.3	13.0		50.0	33.3
19.0	59.2		7.9	2.2		32.2	33.0
6.7	11.4		15.7	16.9		30.1	53.2
4.6	32.9		12.8	12.8		18.2	28.0
8.8	33.3		5.3	7.7		12.5	9.9
6.4	30.6		56.1	29.1		23.1	28.6
7.6	20.2					8.8	27.7
10.6	39.0					10.7	31.5
8.0	23.3					12.5	34.4
3.6	18.7					19.6	49.3
						14.3	31.9
