# Supplementary material for: Cytotoxic Function and Cytokine Production of Natural Killer Cells and Natural Killer T-Like Cells in Systemic Lupus Erythematosis Regulation with Interleukin-15
Source: Mediators Inflamm. 2019 Mar 31;2019:4236562. doi: 10.1155/2019/4236562 (PMC6462338; doi:10.1155/2019/4236562)
Supplement: Supplementary 4 — Figure 3(a): comparison of the MFI of perforin of NK cells from peripheral blood of SLE patients (active and inactive) and healthy controls (normal) in the presence and absence of IL-15. [file 4236562.f4.pdf]

Figure 3(a)

Peforin

Normal			Inactive SLE			Active SLE	
Media	IL-15		Media	IL-15		Media	IL-15
7907	13794		1320	3750		1356	1971
47193	43033		2688	3343		1141	3085
28974	46687		28893	13511		3696	5156
4633	9381		45533	50261		8806	15484
6031	11215		4530	7465		2600	4048
16045	19453		6868	13586		9080	15237
17308	21536		8567	21894		6940	13517
11337	11902		1085	2259		6629	10337
3368	8010		4069	5662		30055	33586
4529	10071		5225	19227		13157	21069
25732	39854		39260	57865		7818	3791
13631	19313		11791	14888		5188	3790
18757	22352		2168	2030		9106	21100
14836	10875		8921	18178		10473	23782
19991	28590					17173	23796
10655	18211					14441	24138
14948	23956					6219	10661
