# Supplementary material for: Cytotoxic Function and Cytokine Production of Natural Killer Cells and Natural Killer T-Like Cells in Systemic Lupus Erythematosis Regulation with Interleukin-15
Source: Mediators Inflamm. 2019 Mar 31;2019:4236562. doi: 10.1155/2019/4236562 (PMC6462338; doi:10.1155/2019/4236562)
Supplement: Supplementary 5 — Figure 3(b): comparison of the MFI of granzyme B of NK cells from peripheral blood of SLE patients (active and inactive) and healthy controls (normal) in the presence and absence of IL-15. [file 4236562.f5.pdf]

Figure 3(b)

Granzyme B

Normal			Inactive SLE			Active SLE	
Media	IL-15		Media	IL-15		Media	IL-15
962	1014		1194	2426		789	857
897	1368		785	995		918	1183
764	1102		1314	1465		797	887
769	921		2180	2443		740	832
615	674		1139	1407		1071	1440
660	641		807	1033		1078	1791
722	704		919	2058		1416	2054
649	682		678	667		2011	2234
1303	1558		553	622		1568	1545
764	941		602	1258		14947	27714
1083	1518		1093	1482		15102	24458
1092	1254					9084	15152
809	1033					13486	16370
1374	1603					7088	38841
						9016	13956
