# Supplementary material for: Cytotoxic Function and Cytokine Production of Natural Killer Cells and Natural Killer T-Like Cells in Systemic Lupus Erythematosis Regulation with Interleukin-15
Source: Mediators Inflamm. 2019 Mar 31;2019:4236562. doi: 10.1155/2019/4236562 (PMC6462338; doi:10.1155/2019/4236562)
Supplement: Supplementary 6 — Figure 4(a): comparison of the percentages of IFN-γ expressing NK cells among normal controls (normal), SLE patients with inactive disease (inactive SLE), and SLE patients with active disease (active SLE) in the presence and absence of IL-15. [file 4236562.f6.pdf]

Figure 4(a)

IFN-gamma

Normal			Inactive SLE			Active SLE	
Media	IL-15		Media	IL-15		Media	IL-15
85.5	88.8		62.5	66.5		75.6	84.7
60.7	52.4		57.4	64		89.9	92.8
74.7	72.5		80.1	85.1		97.5	99.2
61.2	68.1		43.7	59.9		94.7	98.7
58	89.9		56.8	65.1		61.9	64.4
78	76.0		82.7	88.4		52.5	48
50.0	61.3		75.8	88.8		82.3	88.2
60.4	63.2		79	87.3		89.2	93.4
85.5	87.2		81.2	91		76.5	82.4
78.3	70.3		63.8	68.9		88.3	89.2
58.4	61.5					66.3	74.8
55.3	61.5						
60.7	69.4						
66.0	76.7						
55.4	68.8						
73.2	83.4						
81.1	87.5						
