# Supplementary material for: Cytotoxic Function and Cytokine Production of Natural Killer Cells and Natural Killer T-Like Cells in Systemic Lupus Erythematosis Regulation with Interleukin-15
Source: Mediators Inflamm. 2019 Mar 31;2019:4236562. doi: 10.1155/2019/4236562 (PMC6462338; doi:10.1155/2019/4236562)
Supplement: Supplementary 7 — Figure 4(b): comparison of the percentages of TNF-α expressing NK cells among normal controls (normal), SLE patients with inactive disease (inactive SLE), and SLE patients with active disease (active SLE) in the presence and absence of IL-15. [file 4236562.f7.pdf]

Figure 4(b)

TNF-alpha

Normal			Inactive SLE			Active SLE	
Media	IL-15		Media	IL-15		Media	IL-15
74.7	84.6		73.5	86.4		21.4	59.8
74.3	83.2		67.6	78.2		28.3	42
66.2	67.1		81.1	87.6		29.7	65.7
50.7	51.2		67.4	80.7		27.6	54.4
73.9	79.5		88.1	92.9		62.3	70.3
66.7	88.5		67.6	72.1		57.6	73.1
71.8	74.8					41.8	55.8
68.8	72.8					70.7	86.8
76	64.4					34.8	68.7
74.6	64.9					80.5	87
66.4	83.6					73.1	76.4
76.1	81.5						
64.5	84						
91.3	95						
86.3	90.4						
86.5	93.1						
