# Supplementary material for: Cytotoxic Function and Cytokine Production of Natural Killer Cells and Natural Killer T-Like Cells in Systemic Lupus Erythematosis Regulation with Interleukin-15
Source: Mediators Inflamm. 2019 Mar 31;2019:4236562. doi: 10.1155/2019/4236562 (PMC6462338; doi:10.1155/2019/4236562)
Supplement: Supplementary 8 — Figure 5(a): comparison of the MFI of perforin of NKT-like cells from peripheral blood of SLE patients (active and inactive) and healthy controls (normal) in the presence and absence of IL-15. [file 4236562.f8.pdf]

Figure 5(a)

Perforin

Normal			Inactive SLE			Active SLE	
Media	IL-15		Media	IL-15		Media	IL-15
1850	3000		1316	4652		2784	4741
3296	10221		3006	5658		5991	6592
760	820		4809	5393		1871	4894
1083	2410		2176	3810		6799	11369
1511	3225		1102	2479		6180	10584
1468	5011		9196	10973		2369	7724
2557	5390		2020	10638		11314	16989
2825	6639		1465	2808		1145	1859
6711	9418		2423	5368		8792	18361
6971	12085		5713	11756		5136	3056
1795	4988		6850	1758		10183	12478
1992	4913		3553	5298		5145	7664
			5024	9742		8637	12037
						10336	13041
