# Supplementary material for: Cytotoxic Function and Cytokine Production of Natural Killer Cells and Natural Killer T-Like Cells in Systemic Lupus Erythematosis Regulation with Interleukin-15
Source: Mediators Inflamm. 2019 Mar 31;2019:4236562. doi: 10.1155/2019/4236562 (PMC6462338; doi:10.1155/2019/4236562)
Supplement: Supplementary 9 — Figure 5(b): comparison of the MFI of granzyme B of NKT-like cells from peripheral blood of SLE patients (active and inactive) and healthy controls (normal) in the presence and absence of IL-15. [file 4236562.f9.pdf]

Figure 5(b)

Granzyme B

Normal			Inactive SLE			Active SLE	
Media	IL-15		Media	IL-15		Media	IL-15
740	1014		1031	1856		1761	1863
989	1563		830	1088		1067	869
976	1621		1077	1295		856	1120
972	1116		1712	1864		1079	1108
715	692		719	1481		1367	965
727	818		617	756		875	971
900	670		954	1275		1782	1963
690	712		898	1153		2173	1443
990	1311		840	1039		1370	1636
1054	1605		548	1481		1069	1403
1186	1229		705	872		994	1145
729	936		957	830		1114	1128
			1172	1249		883	1089
			861	852		1185	1290
			779	968		1673	1460
						1100	1474
						2759	1832
						2548	2521
						1051	1785
						1201	1159
