# Supplementary material for: Cytotoxic Function and Cytokine Production of Natural Killer Cells and Natural Killer T-Like Cells in Systemic Lupus Erythematosis Regulation with Interleukin-15
Source: Mediators Inflamm. 2019 Mar 31;2019:4236562. doi: 10.1155/2019/4236562 (PMC6462338; doi:10.1155/2019/4236562)
Supplement: Supplementary 10 — Figure 6(a): comparison of the percentages of IFN-γ expressing NKT-like cells among normal controls (normal), SLE patients with inactive disease (inactive SLE), and SLE patients with active disease (active SLE) in the presence and absence of IL-15. [file 4236562.f10.pdf]

Figure 6(a)

IFN-gamma

Normal			Inactive SLE			Active SLE	
Media	IL-15		Media	IL-15		Media	IL-15
64.7	71		57.8	63.4		95.1	96.4
70.9	39.2		73.7	70.3		86.5	84.7
75.3	22.3		71.8	67.9		98.5	98.6
75.8	88.2		71.4	92.4		85	92.6
88	75.3		67.6	76.7		63.5	70.1
61	73.9		88.2	88.2		44.9	60.6
76.6	92.1		69	84.5		88.9	92.6
85.2	92		78.2	96		82.1	88.2
60.6	61.4		71.6	84.8		58.7	83.5
53	76.1					80.8	86.3
69	79.8					44.9	31
58.3	89.3					88.5	91.1
86.4	92.8					83.4	86.7
86	88						
