# Supplementary material for: Cytotoxic Function and Cytokine Production of Natural Killer Cells and Natural Killer T-Like Cells in Systemic Lupus Erythematosis Regulation with Interleukin-15
Source: Mediators Inflamm. 2019 Mar 31;2019:4236562. doi: 10.1155/2019/4236562 (PMC6462338; doi:10.1155/2019/4236562)
Supplement: Supplementary 11 — Figure 6(b): comparison of the percentages of TNF-α expressing NKT-like cells among normal controls (normal), SLE patients with inactive disease (inactive SLE), and SLE patients with active disease (active SLE) in the presence and absence of IL-15. [file 4236562.f11.pdf]

Figure 6(b)

TNF-alpha

Normal			Inactive SLE			Active SLE	
Media	IL-15		Media	IL-15		Media	IL-15
87.7	90.9		64.1	88.2		63.2	71.2
84.4	89.4		76.7	77.5		38.9	51.6
75.9	91.4		88.6	87.7		55.3	88
87.3	85		72.5	83.9		66.8	59.1
84	91.6		81.1	87.6		84.7	90.6
89.5	93		75.6	84.8		68.5	83.3
84.6	77.4					58.8	83.2
80.2	61.5					74.3	88
77.4	91.8					36.1	62.4
87.4	92.6					86.9	90.5
90.2	97.1					89.1	87.9
95.7	95.6						
94.5	95.3						
95.8	96.8						
