# Supplementary material for: Cytotoxic Function and Cytokine Production of Natural Killer Cells and Natural Killer T-Like Cells in Systemic Lupus Erythematosis Regulation with Interleukin-15
Source: Mediators Inflamm. 2019 Mar 31;2019:4236562. doi: 10.1155/2019/4236562 (PMC6462338; doi:10.1155/2019/4236562)
Supplement: Supplementary 12 — Figure 7(a): comparison of the percentages of CD107a expressing NK cells following contact with K562 cells among normal controls (normal), SLE patients with inactive disease (inactive SLE), and SLE patients with active disease (active SLE) in the presence and absence of IL-15. [file 4236562.f12.pdf]

Figure 7(a)

NK

Normal			Inactive SLE			Active SLE	
Media	IL-15		Media	IL-15		Media	IL-15
15.1	51.3		15.8	51		17.3	29
9.1	48.5		14.1	35.5		26.9	37.6
22.6	46		10.6	32.8		10.9	22.4
7.2	26.8		12.5	26.4		9.3	25.2
5.1	11.1		17.6	31.2		7.9	26
3.8	23.3		1.9	20.9		9.5	12.2
8.2	22.9		18.4	35.8		15.6	12.9
8.2	26.3		18.7	27.4		12.5	20.7
23	19.2		14.7	31.6		5.3	18.4
19.8	26.3		21.8	28.5		19.3	24.6
14.4	36.5		12.5	16.9		16.5	28.2
24.7	22.9		16.7	34.9		17.7	16
			14.3	28.2		2.2	22
			7.5	10.2		20.3	39
						14	29.8
						14.4	18
						3.5	19.7
