# Supplementary material for: Cytotoxic Function and Cytokine Production of Natural Killer Cells and Natural Killer T-Like Cells in Systemic Lupus Erythematosis Regulation with Interleukin-15
Source: Mediators Inflamm. 2019 Mar 31;2019:4236562. doi: 10.1155/2019/4236562 (PMC6462338; doi:10.1155/2019/4236562)
Supplement: Supplementary 14 — Table 2: comparison of the percentages of IFN-γ and TNF-α expressing CD56dim and CD56bright NK cells in healthy controls (normal) and SLE patients with active and inactive disease in the presence and absence of IL-15. [file 4236562.f14.pdf]

IFN-gamma-%

CD56dim					
Normal		Inactive SLE		Active SLE	
Media	IL-15	Media	IL-15	Media	IL-15
85.7	89.2	60.4	66.1	70.4	84.1
61.3	52.5	58.8	64.2	88.8	92.4
75.1	72.9	78.6	85.3	96.9	99.4
61.9	68.7	51.3	65.1	93.3	98.7
57	89.4	57.9	64.8	58.4	64.1
78	75.5	83.1	88.7	51.8	60.7
51.4	61.6	76	89.3	83	88.3
61.3	63.7	79.9	87.3	89.5	93.3
84.8	86.0	82.2	91.1	76.6	81.4
78.4	80.3	63.8	69.3	88.6	89.4
58.2	61.3			66.7	76.5
55.1	61.6				
60.8	69.5				
65.7	75.8				
55.5	68.2				
73.1	82.5				
87.4	93.1				

IFN-gamma-%

CD56bright					
Normal		Inactive SLE		Active SLE	
Media	IL-15	Media	IL-15	Media	IL-15
84	62	62.9	60		100
65.7	77.8	88.9		83.3	100
52.5	67.4	87	98.5	100	100
64.8	65.6	22.6	13.3	100	100
70	94.0	56.7	91.3	81	73.5
75	74.8	89.2	91.7	50	62.2
24.2	48.3	55	68.9	95.7	100
46.3	52.4	71.4	76.9	15	91.7
92.3	98.8	85.7	84.6	92.6	98.6
70.4	81.5	74.2	64.6	100	99
80.0	95.2			61.9	53.7
74.2	81.5				
74.2	79.5				
83.4	94.5				
64.2	94.1				
88.4	98.5				
85.1	95.9				

TNF-alpha-%

CD56dim					
Normal		Inactive SLE		Active SLE	
Media	IL-15	Media	IL-15	Media	IL-15
75.8	85.3	74.3	86.6	14.2	57.8
81.2	83.8	69.5	78.3	26.3	42.8
66.3	66.7	81.7	87.8	28.7	64.3
50.2	51.3	68	81.4	25.2	54.6
74.1	77.6	88.7	93	62	69.9
67	88.9	67.6	72.3	58.4	73.6
72.2	74.9			49.4	61
68.9	72.9			71.5	86.9
76	64.4			35.7	68.8
74.8	65.1			81.3	86.7
66.5	83.4			62.9	78.1
76.3	81.4				
64.8	83.4				
91.5	94.4				
86	89.7				
87.4	92.6				

TNF-alpha-%

CD56bright					
Normal		Inactive SLE		Active SLE	
Media	IL-15	Media	IL-15	Media	IL-15
61.9	77.6	40	84	100	100
83.9	93	67.5	74.8	83.3	100
70.9	69.9	76.7	89.7	100	100
53.9	38		88.9	100	100
73.2	86.6	76.6	84.6	57.1	79.6
56.5	86.7	74.2	81.9	68.8	80.7
59.5	81.5			23.5	13.3
82.3	91.1			71.4	92.6
76.9	80.8				100
69.8	73.3			71.4	76.9
73.6	93.7			61.9	53.7
71.1	93				
79.2	94.9				
90.1	97.8				
85.6	92.4				
79.8	95.2				
